# Forgotten joint score is worse when the affected leg perceived longer than shorter after total hip arthroplasty

**DOI:** 10.1186/s12891-023-06573-w

**Published:** 2023-05-31

**Authors:** Takehiro Kawakami, Takashi Imagama, Yuta Matsuki, Tomoya Okazaki, Takehiro Kaneoka, Takashi Sakai

**Affiliations:** Department of Orthopedic Surgery, Yamaguchi University Graduate School of Medicine, 1-1-1 Minami-Kogushi Ube, Yamaguchi Prefecture, Ube, 755-8505 Japan

**Keywords:** Leg length discrepancy (LLD), Forgotten joint score (FJS-12), Perceived leg length discrepancy (P-LLD), Radiographic leg length discrepancy (R-LLD), Total hip Arthroplasty (THA)

## Abstract

**Background:**

One of the causes of patient dissatisfaction after total hip arthroplasty (THA) is leg length discrepancy (LLD). Even when radiographic LLD (R-LLD) is within 5 mm, some people perceive the affected side to be longer, while others perceive it is shorter. The purpose of this study was to investigate the relationship between perceived LLD (P-LLD), R-LLD, and Forgotten Joint Score (FJS-12) after THA.

**Methods:**

A retrospective study of 164 patients with unilateral hip disease was conducted. Based on P-LLD after THA, they were classified into three categories: perceived short (PS 21 patients), no LLD (PN 121 patients), and perceived long (PL 22 patients). On the other hand, based on R-LLD after THA, they were divided into <  − 5 mm (RS 36 patients), − 5 mm ≤ x < 5 mm (RN 99 patients), and 5 mm ≥ (RL 29 patients), respectively. The proportion of P-LLD in the RN group was also evaluated. In each group, the relationship between P-LLD, R-LLD and FJS-12 was investigated.

**Results:**

After THA, the PL group had significantly worse FJS-12 (PS: 68.3 ± 26.2, PN: 75.0 ± 20.9, PL: 47.3 ± 25.2, *P* < .0001). In the R-LLD evaluation, there was no difference in FJS-12 among the three groups (RS: 73.7 ± 21.1, RN: 70.0 ± 24.5, RL: 67.7 ± 25.4, *P* < .53). The RN group perceived leg length to be longer (RN-PL) in 12.1% of cases, and the RN-PL groups had significantly worse FJS-12 (RN-PS: 65.4 ± 24.8, RN-PN: 73.8 ± 23.1, RN-PL: 41.8 ± 27.6, *P* < .0001).

**Conclusion:**

One year after THA, patients with longer P-LLD had worse FJS-12, even if the R-LLD was less than 5 mm.

## Introduction

Total hip arthroplasty (THA) is one of the most successful orthopedic surgery procedures [1]. The primary goal of THA is to reduce pain and improve function, but it is also hoped that the led length discrepancy (LLD) will improve. LLD following THA has been linked to poor functional outcome and patient satisfaction [2, 3]. The incidence of LLD following primary THA varies throughout the literature and has been reported to range from 1 to 27%, which was the second most common reason for litigation [2–4]. LLD evaluation includes both perceived LLD (P-LLD) and radiographic LLD (R-LLD).

Behrend developed the Forgotten Joint Score (FJS-12) in 2012 as a patient-reported outcome measure (PROM) to assess prosthesis awareness, and it has been shown to have a low ceiling effect [5]. Forgetting arthroplasty can be viewed as a goal of arthroplasty, and it is thought to maximize patient satisfaction [5]. Poor prognostic factors of FJS-12 after THA include contralateral hip condition, female sex, smoking, and knee joint effects [6–8]. On the other hand, there have been no reports of an association between P-LLD or R-LLD and FJS-12.

We hypothesized that the FJS-12 at 1 year after THA is worse if P-LLD and R-LLD are present. This study was carried out to investigate the relationship between P-LLD, R-LLD and FJS-12 at 1 year after THA.

## Patients and methods

**Fig. 1:**
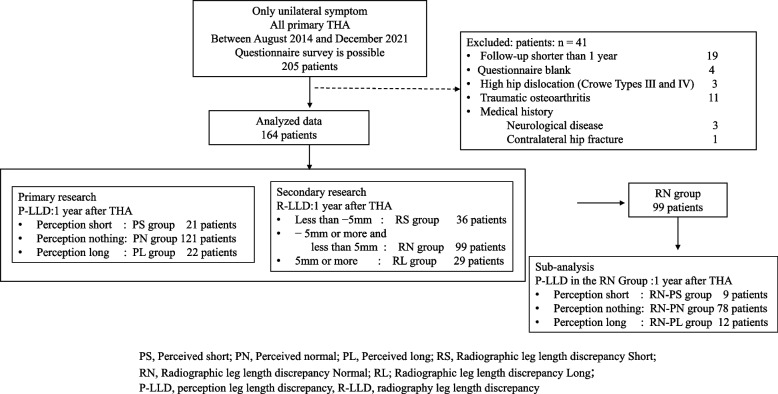
Patient selection flowchart

The study is retrospective study. The study included 205 patients with unilateral hip arthritis who underwent primary THA between April 2014 and December2021. All patients were questioned in a questionnaire one year after THA surgery. (Fig. 1). Exclusion criteria included 19 patients with < 1 year follow-up, 4 patients with blanks on the questionnaire, 3 patients with high hip dislocation (Crowe Type III 2 patients, Type IV, 1 patients), 11 patients with traumatic osteoarthritis, 3 patients with pre-existing neurological diseases, and 1 patient with contralateral hip fracture surgery. As a result, we examined 164 patients (male 26 patients, 138 female patients). This study was performed in line with the principles of the Declaration of Helsinki. The trial protocol was approved by the Ethics Committee and Institutional Review Board of Yamaguchi University Hospital (H2020-068–2) and all patients provided informed consent.

P-LLD and R-LLD were evaluated 1 year after THA. Age, gender, body mass index (BMI), surgical approach, hip disease, global offset (GO), perioperative change in leg length (ΔL), preoperative Cobb angle, preoperative pelvic obliquity, FJS-12, and the Japanese Orthopedic Association Hip Disease Evaluation Questionnaire (JHEQ) satisfaction [9] were also investigated.

### Preoperative planning and surgery

For all patients, preoperative planning was done using a computed tomography (CT)-based three-dimensional templating system (ZedHip; LEXI, Tokyo, Japan), with offset and leg length planned to be aligned with the healthy contralateral side. The surgery was performed by the same team, and the final offset and leg length were determined based on preoperative planning. When lumbar scoliosis was observed, preoperative anteroposterior lumber X rays were imaged with right and left lateral flexion to investigate lumber stiffness of the lumber scoliosis. Due to pelvic obliquity and stiffness of lumber scoliosis, we determined the preoperative planning not to make perceived leg length discrepancy. However, when cases that intraoperative joint instability were concerned, leg lengthening was performed minimally.

### Radiographic analysis

**Fig. 2:**
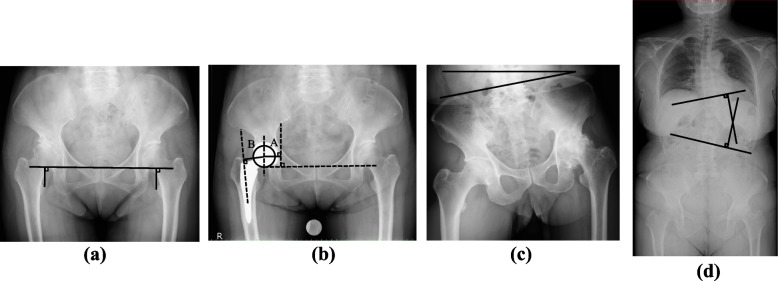
Radiographic analysis

Radiographic analysis was taken by one author (T.K) not involved in the surgery. Anterior–posterior (AP) radiographs were taken in the supine position before surgery and 1-year after surgery. The X-ray beam was focused on the pubic symphysis. Further, R-LLD was measured at the lesser trochanter’s apex using the lower margin of the teardrop as a reference line, which is reproducible on the pelvis [10, 11] (Fig. 2a). R-LLD defined the difference between the teardrop and lesser trochanter as measured on the operative vs non-operative side. The measurements were recorded to the nearest 1 mm. Preoperative and 1-year postoperative measurements were taken. The perioperative change in leg length (ΔL) was defined as the difference between preoperative R-LLD and postoperative R-LLD.

The offset was evaluated the global offset (GO). GO was measured as the sum of femoral offset (FO) and acetabular offset (AO) [12, 13]. FO, defined as the perpendicular distance from the center of rotation of the femoral head to the anatomical femoral axis, was measured on AP pelvis radiographs both preoperatively and postoperatively (Fig. 2b). On the other hand, AO, defined as the perpendicular distance from the center of rotation of the femoral head to the line passing through the medial edge of the ipsilateral teardrop perpendicular to the line passing through the lower margins of the ischial tuberosity, was measured on AP pelvis radiographs both preoperatively and postoperatively. GO was measured preoperatively and 1-year postoperatively as the healthy side ratio (affected side GO/healthy side GO) and the healthy side difference (affected side GO − healthy side GO).

The angle between the bilateral iliac crests and the horizontal reference line (a line drawn parallel to the floor) was used to calculate pelvic obliquity [14] (Fig. 2c). Based on previous reports, preoperative standing pelvic AP X-rays were used for measurement, [15].

Standing frontal preoperative X-rays were used to calculate Cobb angle. (Fig. 2d) The curve is calculated by identifying the vertebral bodies at the superior and inferior margins of the curve (also known as the terminal vertebral bodies) [16]. Tangent lines are drawn from the superior end plate of the superior vertebra and the inferior end plate of the inferior vertebra.

### Patient-Reported Outcome Measure (PROM)

**Fig. 3:**
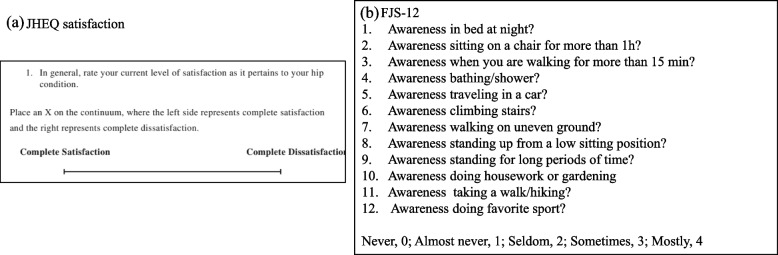
Patient-Reported outcome measures

One year after THA, the JHEQ, FJS-12, and P-LLD questionnaires were administered in all patients. In the JHEQ satisfaction (Fig. 3a), dissatisfaction with the patient’s current condition on each side is marked on a visual analog scale (VAS) of 0 mm (complete satisfaction) to 100 mm (complete dissatisfaction). Moreover, the FJS-12 is made up of 12 questions (Fig. 3b), each with a five-point Likert response format, and is scored from 0 to 48. The raw score is normalized to a range of 0 (worst condition) to 100 points (best condition). In previous reports, Using satisfaction as the anchor, the Minimal clinically important difference (MCID) for the FJS was 8.1, which was affirmed when adjusting for confounding [17]. The patients were evaluated for P-LLD 1 year after THA by completing the following questionnaire: Q1: Do either of your legs feel longer now? Yes or no? Q2: If you answered “yes” to Q1, which leg is it? Right or left﻿?

### Statistics analysis

In this study, data analysis was carried out using JMP ® Pro 15 (SAS Institute Inc.). The statistical significance of the differences between the three groups was determined using ANOVA. A post-hoc test was performed for those in which a significant difference was recognized. For the statistics of nominal variables, the chi-square test was used. Statistical significance was assigned for values of *P* < 0.05. Moreover, statistical data are presented as means and standard deviations.

## Results

In the P-LLD evaluation, 21 patients were assigned to the group that perceived shorter leg length (PS group), 121 patients to the group that perceived no leg length discrepancy (PN group), and 22 patients to the group that perceived longer leg length (PL group) (Fig. 1). There were no significant differences among the three groups in terms of age, gender, BMI (kg/m^2^), surgical approach, preoperative GO, or disease (Table 1). The PS group had shorter preoperative and postoperative R-LLD than the PN and PL groups (Preoperative R-LLD: PS: -15.3 ± 1.3, PN: -8.8 ± 0.8, PL: -4.9 ± 1.5, *P* < 0.0001, Postoperative R-LLD: PS: -7.5 ± 9.5, PN: -0.2 ± 5.5, PL: -0.5 ± 5.8, *P* < 0.0001).

**Table 1:** Patient characteristics in P-LLD evaluation

	PS Group	PN Group	PL Group	*P* value
	(*n* = 21)	(*n* = 121)	(*n* = 22)	
Age (years)	66.1 ± 12.4	65.6 ± 11.3	69.4 ± 10.0	0.37
Gender (%)				0.6
Male	4 (19.1)	20 (16.5)	2 (9.1)	
Female	17 (81.0)	101 (83.5)	20 (90.9)	
BMI (kg/m^2^)	24.2 ± 4.5	24.0 ± 4.8	22.9 ± 4.5	0.69
Approach (%)				0.83
PL	9 (42.9)	54 (48.1)	7 (31.8)	
mWJ	2 (9.5)	8 (6.6)	2 (9.1)	
DAA	10 (47.6)	59 (48.8)	13 (59.1)	
Preoperative R-LLD	-15.3 ± 1.3^a,b^	-8.8 ± 0.8^a^	-4.9 ± 1.5^b^	< .0001
Postoperative R-LLD	-7.5 ± 9.5^a,b^	-0.2 ± 5.5^a^	-0.5 ± 5.8^b^	< .0001
Preoperative GO				
Healthy side rate	1.0 ± 0.2	1.0 ± 0.1	1.0 ± 0.1	0.29
Healthy side difference (mm)	0.8 ± 12.0	-0.7 ± 6.1	1.4 ± 6.2	0.33
Diagnosis (%)				0.50
Primary OA	10 (47.6)	70 (57.9)	10 (45.5)	
Secondary OA	9 (42.9)	35 (28.9)	8 (36.4)	
ONFH	2 (9.5)	9 (7.4)	3 (13.6)	
RDC	0 (0.0)	6 (5.0)	0 (0.0)	
SIF	0 (0.0)	1 (0.8)	1 (4.6)	

^*P*^^*S*^^Perceived short, *PN* Perceived normal, *PL* Perceived long^

^*BMI*^^Body mass index, *PL* Postlateral, *mWJ* modified Watson Jones, *DAA* Direct anterior approach^

^a^ *P* < 0.05, ^b^ *P* < 0.05

**Fig. 4:**
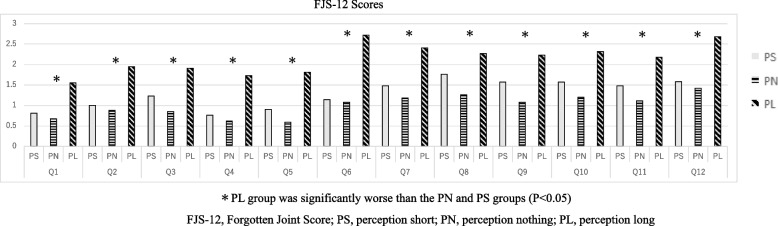
Comparison Between Three Groups by The FJS-12 Question in P-LLD Evaluation

Among three groups distributed based on the P-LLD evaluation, there were no significant differences in ΔL, healthy side ratio of postoperative GO, and healthy side difference, except for FJS-12 (Table 2). The FJS-12 1 year after THA was significantly lower in the PL group compared to the PN and PS groups (PS: 68.3 ± 26.2, PN: 75.0 ± 20.9, PL: 47.3 ± 25.2, *P* < 0.0001). On the other hand, JHEQ satisfaction did not differ significantly among the three groups (PS: 91.2 ± 12.2, PN: 90.5 ± 16.5, PL: 83.6 ± 22.9, *P* = 0.20) (Table 2). On all items of FJS-12, the PL group performed significantly worse than the PN and PS groups (Fig. 4).

**Table 2:** Postoperative offset and the FJS-12 and JHEQ satisfaction in P-LLD evaluation

	PS Group	PN Group	PL Group	*P* value
	(*n* = 21)	(*n* = 121)	(*n* = 22)	
ΔL(mm)	9.8 ± 9.6	8.4 ± 7.6	8.1 ± 7.1	0.73
Postoperative GO				
Healthy side rate	1.0 ± 0.1	1.0 ± 0.1	1.0 ± 0.1	0.80
Healthy side difference (mm)	1.3 ± 9.4	2.8 ± 6.9	1.8 ± 8.4	0.64
FJS-12	68.3 ± 26.2^a^	75.0 ± 20.9^b^	47.3 ± 25.2^a,b^	< .0001
JHEQ satisfaction	91.2 ± 12.2	90.5 ± 16.5	83.6 ± 22.9	0.20

*PS* Perceived short, *PN* Perceived normal, *PL* Perceived long

ΔL: Difference in preoperative and postoperative measured radiography leg length difference

*GO* Global offset, *FJS-12* Forgotten Joint Score

*JHEQ* Japanese Orthopaedic Association Hip Disease Evaluation Questionnaire

^a^*P* < 0.05.^b^ *P* < 0.05

In the P-LLD evaluation, preoperative Cobb angle was significantly greater in the PL and PS groups (Table 3). In addition, the PS group had a significantly higher preoperative pelvic obliquity angle. The proportions of Lumber Scoliosis (Normal: Affected side: Healthy side) in the P-LLD groups including PS, PN, and PL groups were PS (9:10:2), RN (72:33:17), and RL (4:11:7), respectively (*P* = 0.004). The proportions pelvic obliquity (Normal: Tilt affected side: Tilt Healthy side) in the P-LLD groups including PS, PN, and PL groups were PS (10:7:4), RN (80:25:16), and RL (12:9:1), respectively. There was no significant difference in preoperative Pelvic obliquity among the three groups (*P* = 0.15).

**Table 3:** Preoperative cobb angle and pelvic obliquity angle in P-LLD evaluation

	PS Group	PN Group	PL Group	*P* value
	(*n* = 21)	(*n* = 121)	(*n* = 22)	
Cobb angle	10.5 ± 10.2^a^	6.2 ± 7.1^a^^,b^	11.0 ± 7.4^b^	0.004
Lumber Scoliosis (%)				0.004
Normal	9 (42.9)	72 (59.5)	4 (18.8)	
Affected side	10 (47.6)	33 (27.3)	11 (50.0)	
Healthy side	2 (9.5)	17 (14.0)	7 (31.8)	
Pelvic obliquity angle	4.6 ± 3.6^a,b^	2.3 ± 2.0^a^	2.3 ± 1.8^b^	0.001
Pelvic obliquity (%)				0.15
Normal	10 (47.6)	80 (66.1)	12 (54.6)	
Tilt affected side	7 (33.3)	25 (20.7)	9 (40.9)	
Tilt healthy side	4 (19.5)	16 (13.2)	1 (4.6)	

*PS* Perceived short, *PN* Perceived normal, *PL* Perceived long

^a^ *P* < 0.05, ^b^ *P* < 0.05

One year after THA, In the R-LLD evaluation, 36 patients were distributed to the group with a radiography leg length discrepancy of less than -5 mm (RS group), 99 patients to the group with more than -5 mm and less than 5 mm (RN group), and 29 patients to the group with more than 5 mm longer (RL group) (Fig. 1). There were no significant differences between the three groups in terms of age, sex, BMI (kg/m^2^), surgical approach, preoperative GO, or disease (Table 4). The RS group had shorter preoperative R-LLD than the RN and RL groups (RS: -16.8 ± 1.8, RN: -0.4 ± 3.1, RL: 7.2 ± 3.5, *P* < 0.0001).

**Table 4:** Patient characteristics in R-LLD evaluation

	RS Group	RN Group	RL Group	*P* value
	(*n* = 36)	(*n* = 99)	(*n* = 29)	
Age (years)	66.0 ± 12.2	66.3 ± 10.8	66.3 ± 12.3	0.99
Gender (%)				0.93
Male	5 (13.9)	16 (16.2)	5 (17.2)	
Female	31 (86.1)	83 (83.8)	24 (82.8)	
BMI (kg/m^2^)	25.2 ± 4.5	23.4 ± 3.8	23.2 ± 7.0	0.17
Approach (%)				0.47
PL	15 (41.7)	44 (44.4)	11 (37.9)	
mWJ	5 (13.9)	6 (6.1)	1 (3.5)	
DAA	16 (44.4)	49 (49.5)	17 (58.6)	
Preoperative R-LLD	-16.8 ± 1.8^a^^,b^	-8.6 ± 0.7^a^	-7.6 ± 1.7^b^	< .0001
Postoperative R-LLD	-9.5 ± 6.1^a,b^	-0.4 ± 3.1^a^	7.2 ± 3.5^b^	< .0001
Preoperative GO				
Healthy side rate	1.0 ± 0.1	1.0 ± 0.1	1.0 ± 0.1	0.33
Healthy side difference	-1.7 ± 9.8	0.5 ± 5.7	-0.8 ± 7.6	0.25
Diagnosis (%)				0.29
Primary OA	19 (52.8)	51 (51.5)	20 (69.0)	
Secondary OA	15 (41.7)	32 (32.3)	5 (17.2)	
ONFH	1 (3.0)	9 (9.1)	4 (13.8)	
RDC	1 (3.0)	5 (5.1)	0 (0.0)	
SIF	0 (0.0)	2 (2.0)	0 (0.0)	

*RS* Radiographic leg length discrepancy Short, *RN* Radiographic leg length discrepancy Normal,

*RL* Radiographic leg length discrepancy Long, *PL* Postlateral, *mWJ* modified Watson Jones, *DAA* Direct anterior approach, *GO* Global offset, *OA* Osteoarthritis, ONFH

^a^ *P* < 0.05, ^b^ *P* < 0.05

One year after THA, among the three groups, ΔL was significantly larger in the RL group than in the RS and RN groups (PS: 6.1 ± 8.9, PN: 8.4 ± 7.7, PL: 12.0 ± 5.1, *P* = 0.01) (Table 5). There was no significant difference in the healthy side ratio or healthy side difference in postoperative GO. The FJS-12 was not different between the three groups 1 year after THA (PS: 73.7 ± 21.1, PN: 70.0 ± 24.5, PL: 67.7 ± 25.4, *P* = 0.53). The JHEQ satisfaction also did not differ significantly among the three groups (PS: 88.8 ± 19.1, PN: 90.2 ± 16.9, PL: 89.1 ± 15.4, *P* = 0.10).

**Table 5:** Postoperative offset and the FJS-12 and JHEQ Satisfaction in R-LLD evaluation

	RS Group	RN Group	RL Group	*P* value
	(*n* = 36)	(*n* = 99)	(*n* = 29)	
ΔL (mm)	6.1 ± 8.9^a^	8.4 ± 7.7^b^	12.0 ± 5.1^a^^,b^	0.01
Postoperative GO				
Healthy side rate	1.0 ± 0.1	1.0 ± 0.1	1.1 ± 0.1	0.34
Healthy side difference (mm)	0.7 ± 8.6	2.9 ± 7.1	3.3 ± 7.0	0.25
FJS-12	73.7 ± 21.1	70.0 ± 24.5	67.7 ± 25.4	0.53
JHEQ satisfaction	88.8 ± 19.1	90.2 ± 16.9	89.1 ± 15.4	0.10

The proportions of P-LLD (PS:PN:PL) in the R-LLD groups including RS, RN, and RL groups were RS (11:20:5), RN (9:78:12), and RL (1:23:5), respectively. In the sub-analysis in the RN group, FJS-12 was significantly lower in the RN-PL group (RN-PS: 65.4 ± 24.8, RN-PN: 73.8 ± 23.1, RN-PL: 41.8 ± 27.6, *P* < 0.0001) and there was no significant difference in the JHEQ satisfaction (RN-PS: 94.6 ± 7.2, RN-PN: 90.1 ± 17.6, RN-PL: 81.0 ± 27.5, *P* = 0.10) (Table 6). When the three groups were compared for each of the 12 items on the FJS-12, the RN-PL group performed significantly worse than the RN-PS and RN-PN groups on all items.

**Table 6:** Postoperative FJS-12 and JHEQ Satisfaction sub-analysis

	RN-PS Groups	RN-PN Groups	RN-PL Groups	*P* value
	(*n* = 9)	(*n* = 78)	(*n* = 12)	
FJS-12 (range)	65.4 ± 24.8^a^ (29.2–100)	73.8 ± 23.1^b^ (18.8–100)	41.8 ± 27.6^a,b^ (0–77)	< .0001
JHEQ satisfaction (range)	94.6 ± 7.2 (80–100)	90.1 ± 17.6 (6–100)	81.0 ± 27.5 (7–100)	0.10

*RN* Radiographic leg length discrepancy Normal, *PS* Perceived short, *PN* Perceived normal, *PL* Perceived long, *FJS-12* Forgotten Joint Score

*JHEQ* Japanese Orthopaedic Association Hip Disease Evaluation Questionnaire

^a^ *P* < 0.05, ^b^ *P* < 0.05

## Discussion

The most important finding of this study was that patients with longer P-LLD 1 year after THA had a worse FJS-12. Even when the R-LLD was within 5 mm, 13.4% of the patients thought the affected side was longer, indicating that the FJS-12 was worse. Although previous reports have shown that P-LLD reduces functional outcome and patient satisfaction after THA [2, 3], the previous study did not mention that longer P-LLD was worse than shorter P-LLD. The present study is valuable to us because it elucidated the relationship between longer P-LLD and worse FJS-12.

In the present study, the FJS-12 scores of the RN-PL groups were significantly lower 1 year after THA. In the RN group, the percentage was higher in the RN-PL group (12.1%) than in the RN-PS group (9.1%). According to Konyves et al., longer leg length is more likely to be recognized than shorter leg length after THA [18]. In addition, Friberg et al. reported that approximately 80% of unilateral hip arthropathy patients with sciatic symptoms had symptoms in the leg that were perceived longer and that correcting the leg length inequality with an adequate shoe lift and P-LLD improved the sciatic symptoms [19]. These findings indicate that there are many cases where patients have perceived leg length discrepancy and believed that the affected side is longer than the other side, even when there is little difference in radiography leg length. In addition, P-LLD has been linked to chronic back pain, the need for supplemental height, claudication, and other adverse effects in patients [20, 21]. In the present study, the PL and RN-PL groups performed worse than the PS and PN groups on all FJS-12 items, indicating that they are negatively affected in various daily life situations, such as resting, walking, and standing.

The PL groups performed poorly on the FJS-12, but there was no statistically significant difference in the JHEQ satisfaction among the three groups. The VAS rates the JHEQ satisfaction as a single item, whereas the FJS-12 rates 12 daily activities. These satisfaction surveys may not be exactly the same. When patients who could not walk before the surgery improved to walk 200–300 steps per day postoperative, the satisfaction level is high but FJS-12 is low. Patient satisfaction level may be different from FJS-12 evaluation. Although more detailed research is necessary to investigate differences scientifically, this is beyond the purpose of the present study, which focuses on leg length discrepancy.

The preoperative risk factors for P-LLD after THA include pelvic obliquity, lumbar spine mobility, difference in knee flexum/recurvatum angle, and difference in distance between the middle of the tibial plafond and the ground [22, 23]. In the present study, the PL and PS groups had larger preoperative Cobb angles than the PN group. On the other hand, the PS group had a larger preoperative pelvic obliquity angle than the PL and PN groups. Based on the results of this study, it is considered necessary that attention should be paid to preoperative scoliosis in the future. In the present study, PL group was a poor factor for postoperative FJS-12. There was no significant difference in R-LLD between PL group and PN group before and after the surgery. On the other hand, scoliosis was more frequent in PL group than in PN group. In cases of scoliosis with small preoperative leg length difference, preoperative planning should be done to ensure that leg lengthening is limited minimally on the affected side. In the preoperative plan, the postoperative leg length difference should be within 5 mm compared to the affected side. However, this is retrospective study and may be biased in its examination of risk factors for P-LLD. In present study, it is clear that in order to improve the FJS-12 the risk factors of PL should be considered, not the risk factors of PS.

According to Flecher et al., increasing offset after THA can cause excessive muscle tension, pelvic lateral tilt, and P-LLD [11]. On the other hand, Zhang et al. reported that pelvic obliquity gradually improved 1 year after THA, as did P-LLD [24]. In the present study, there was no significant difference in the healthy side rate or difference in the healthy side of postoperative GO among the PS, PN, and PL groups. In this study, a CT-based three-dimensional templating system was used for preoperative planning, and the offset was planned to be no more than 10 mm compared to the healthy side, so the effect of the offset was considered minor.

P-LLD was involved in the FJS-12, whereas R-LLD was not. P-LLD has been shown to produce an average of 3–17 mm of R-LLD [19.22]. On the other hand, Wylde et al. reported that 30% of patients had P-LLD after THA surgery, but only 36% had R-LLD, making assessing P-LLD on imaging studies difficult [25]. Lazennec et al. also reported that approximately 50%–60% of patients have P-LLD regardless of the difference in anatomic femoral length between the operative and nonoperative sides, and this is true even when the difference is only 1 mm [20]. In the present study, 25.6% had a perceived leg length discrepancy 1 year after THA, with 57.1% having an R-LLD of less than 5 mm. These findings imply that evaluating P-LLD by R-LLD is difficult and that R-LLD was not involved in the FJS-12.

In the R-LLD evaluation, the RS group had shorter preoperative R-LLD than the RN and RL groups. Also ΔL was significantly larger in the RL group than in the RS and RN groups). In the preoperative planning, the postoperative leg length difference is within 5 mm compared to the affected side, but in some cases where intraoperative joint instability was a concern, leg lengthening ≥ 5 mm was performed. Therefore, it is thought that ΔL increased in the RL group.

This retrospective study has some limitations. First, there is a possibility that loss of follow-up and selection bias will be identified. Second, the condition of the knee joint could not be evaluated and the total length of the lower extremities could not be evaluated in some cases. Thirdly, although preoperative planning was done in three-dimensional planning, postoperative offset and leg length were evaluated only by plain radiographs. Tamura et al. examined femoral length in patients with unilateral hip osteoarthritis or developmental dysplasia of the hip on CT and found potential asymmetry [26]. In the future, it will be necessary to investigate the impact of femoral length and knee joint on R-LLD. However, R-LLD and P-LLD were also underrepresented in previous reports [20, 26]. We believe that this study is important because it is the first to suggest that the perception of longer leg length discrepancy is a risk factor for the FJS-12.

## Conclusion

One year after THA, patients with longer P-LLD had worse FJS-12. Furthermore, 12.6% of the patients had longer P-LLD even if the R-LLD was less than 5 mm, and similarly, FJS-12 was worse.

## Data Availability

The datasets used and/or analysed during the current study available from the corresponding author on reasonable request.
